# Bone Marrow Infiltration Is a Distinctive Risk Factor for Rituximab Infusion-Related Reactions in CD20-Positive B-Cell Non-Hodgkin Lymphoma

**DOI:** 10.1155/2022/3688727

**Published:** 2022-02-11

**Authors:** Shinya Ohata, Kei Takenaka, Daisuke Sugiyama, Takeshi Sugimoto

**Affiliations:** ^1^Department of Hematology and Oncology, Kita-Harima Medical Center, Ono, Hyogo, Japan; ^2^Department of Oncology and Hematology, Kobe University Hospital, Kobe, Hyogo, Japan; ^3^Faculty of Nursing and Medical Care, Keio University, Tokyo, Kanagawa, Japan

## Abstract

**Background:**

Bone marrow infiltration of lymphoma cells is a candidate risk factor for infusion-related reactions (IRRs) in patients with CD20-positive B-cell non-Hodgkin lymphoma (B-NHL). However, despite with the lack of sufficient data, the effect of bone marrow infiltration of B-NHL cells on the incidence rate of grade 2 or higher IRRs with the administration of rituximab has been retrospectively studied in this paper.

**Methods:**

Patients with CD20-positive B-NHL who received the rituximab induction therapy for the first time were enrolled in this study. To evaluate the bone marrow infiltration of B-NHL cells, May–Giemsa stain of bone marrow films and flow cytometry examination of bone marrow aspiration samples were performed. IRR grade was determined using the IRR criteria in the Common Terminology Criteria for Adverse Events version 4.0.

**Results:**

A total of 127 patients were eligible for this study. Grade 2 or higher IRRs were observed in 43 (34%) patients. In univariate analysis, use of glucocorticoid before rituximab infusion was a strong risk-avoiding factor for grade 2 or higher IRRs. Advanced stage of disease (Ann Arbor: stages III and IV) or bone marrow infiltration of B-NHL cells revealed the risk factors, regardless of glucocorticoid premedication. Using multivariate analysis, bone marrow infiltration was found to be an independent risk factor for patients without prior glucocorticoid use.

**Conclusion:**

Bone marrow infiltration of B-NHL cells is a risk factor for grade 2 or higher IRRs at the first rituximab induction therapy without glucocorticoid premedication.

## 1. Introduction

Rituximab is used as a chimeric anti-CD20 monoclonal antibody drug for the human CD20 antigen expressed on the surface of B lymphoma cells or normal B lymphocytes. Rituximab is useful for treating CD20-positive B-cell non-Hodgkin lymphoma (B-NHL) or CD20-positive lymphoproliferative disorders under immunosuppressive treatment [1]. Rituximab can also be used to treat vasculitis, including granulomatosis with polyangiitis and microscopic polyangiitis [2]; immune thrombocytopenic purpura [3]; or refractory nephrotic syndromes [4]. Regarding CD20-positive B-NHL, rituximab has become a standard treatment drug and is applied as the first-line therapy in combination with other cytotoxic drugs. Adverse events associated with rituximab usage are characterized based on the observations following monoclonal antibody therapy. Myelosuppressive reaction or gastrointestinal symptoms are scarce under rituximab treatment compared with treatment with cytotoxic drugs, but rituximab is associated with a risk of emerging infusion-related reactions (IRRs). The symptoms of IRRs include chills, fever, rashes, nausea, asthenia, and headache. IRRs may develop into respiratory and cardiovascular symptoms and anaphylactoid reactions [5]. The detailed mechanism underlying IRRs remains to be clarified; however, the cytokine release syndrome that occurs due to the immunoreaction between lymphocytes and tumor cells may be of concern [6]. In clinical practice, developing IRRs during the second or after the third infusion of rituximab is not frequently recognized, and the reason for reducing the IRR risk comes from the decreased cytokine reaction in accordance with the reduction of CD20-positive B-NHL cells. Therefore, IRR management during rituximab induction therapy is important to maintain the relative dose intensity of the rituximab-containing regimen while also ensuring patient's safety. IRRs have been observed in around 80% of patients during rituximab induction therapy for CD20-positive B-NHL [7]. The majority of the cases is mild; however, ∼10% of cases develop severe reactions, including respiratory failure, hypotension, angioedema, and hypoxia [8]. The potential problems in clinical practice would be grade 2 or higher levels of IRRs that require speedy reduction, interruption of rituximab infusion, and medical response, including glucocorticoid or antihistamine intervention.

According to earlier studies on IRRs in B-cell malignancy, high levels of circulating tumor cells and bulky disease are independent risk factors [6, 9–11]. In terms of the laboratory index of IRR risk factors, increased levels of serum lactate dehydrogenase (LDH) to more than the upper normal limit or of serum interleukin-2 receptor (sIL-2R) to >2000 IU/L have been reported [12], in which case, both indices might reflect high tumor volume. Indolent lymphoma [13] or B symptoms [14] are also independent risk factors for IRRs. Several retrospective studies have reported that bone marrow infiltration of lymphoma cells appears to be a risk factor for IRRs [10, 13, 15], but conclusive evidence remains insufficient. The effect of bone barrow infiltration of lymphoma cells on the incidence rate of grade 2 or higher IRRs at the rituximab induction therapy within CD20-positive B-NHL has been investigated in this study.

## 2. Materials and Methods

### 2.1. Patients and Samples

In this retrospective study, patients admitted to the Department of Hematology and Oncology in Kita-Harima Medical Center (KHMC) between October 2013 and March 2019 were enrolled. The inclusion criteria were as follows: (1) diagnosed with CD20-positive B-NHL; (2) underwent induction chemotherapy with rituximab infusion or a rituximab-containing regimen or relapsed or refractory patients who had not received rituximab before; and (3) underwent bone marrow aspiration testing or bone marrow biopsy within 20 days before rituximab induction therapy. If the bone marrow status for lymphoma infiltration was not known for this study period, the patient was excluded. This study was reviewed and approved by institutional review boards at KHMC (approval no. R02–3; approval date: April 3, 2020).

Using electric medical records (EMRs), patient data were retrospectively collected, including age, gender, height, weight, histopathological subtype of CD20-positive B-NHL according to the World Health Organization classification [16], clinical staging by Ann Arbor classification, B symptoms, chemotherapy regimen, Eastern Cooperative Oncology Group (ECOG) performance status score, and existence of splenomegaly. Laboratory data including hemoglobin levels, circulating lymphoma cells, serum LDH levels, sIL-2R levels, vital signs, and premedication for rituximab infusion including glucocorticoid usage were also collected. The IRR grade in each case was determined using the case description, highest body temperature within 24 h after rituximab infusion, and responsive treatment and prescription contents for IRRs in the EMR.

Rituximab (Zenyaku Kogyo, Tokyo, Japan) was administered as an intravenous infusion at a dose of 375 mg/m^2^ in all cases, and the combination of acetaminophen 400 mg and hydroxyzine 25 mg was taken orally as premedication prior to rituximab infusion. A biosimilar drug for the CD20 monoclonal antibody was not used in this study. Glucocorticoid premedication was not performed; however, it was adapted in some cases to achieve antipyretic effect for tumor fever or to reduce tumor volume before rituximab administration, as per the discretion of the attending physician. Oral prednisolone was administered as a glucocorticoid premedication. If oral glucocorticoid premedication was difficult to administer, drop infusion of prednisolone sodium succinate was given. Rituximab infusion was started at an initial dose of 50 mg/h for 30 min. After confirming the absence of IRRs in a patient, drop infusion speed was increased to 100 mg/h for 30 min and then up to 200 mg/h until the entire volume was finished. When a patient began to present with IRRs, each attending physician decided to stop with or reduce the dose of rituximab infusion and provide subsequent treatment intervention.

### 2.2. Evaluation of Bone Marrow Infiltration with CD20-Positive B-NHL Cells

To determine the bone marrow infiltration of CD20-positive B-NHL cells, a bone marrow film stained with the May–Giemsa method was observed to confirm the presence of abnormal cells, and the clonality of tumor cells was evaluated using flow cytometry (FCM) analysis using CD5, CD10, CD19, CD20, kappa, and lambda antibodies. If a patient had dry tap marrow, bone marrow biopsy specimen by immunochemistry staining with the anti-CD20 antibody as well as anti-CD19, anti-CD10, anti-kappa, and anti-lambda antibodies was used to evaluate lymphoma infiltration. In this study, the result of the positron emission tomography-computed tomography scan was not referred to as an evaluation of bone marrow infiltration of lymphoma cells.

### 2.3. Definition of IRRs

IRRs were defined as reactions that emerged within 24 h after starting rituximab infusion. The level of IRRs in each patient was determined according to the criteria of infusion-related reaction in the Common Terminology Criteria for Adverse Events (CTCAE) version 4.0. The details of these criteria were as follows: grade 1, mild transient reaction (infusion interruption not indicated and intervention not indicated); grade 2, therapy or infusion interruption indicated but responds promptly to symptomatic treatment (e.g., antihistamines, NSAIDs, narcotics, and IV fluids) and prophylactic medication indicated for ≤24 h; grade 3, prolonged (i.e., not rapidly responsive to symptomatic medication and/or brief interruption of infusion), recurrence of symptoms following initial improvement, and hospitalization indicated for other clinical sequelae; grade 4, life-threatening consequences and urgent intervention indicated; and grade 5, death. Clinically significant IRRs were defined as grade 2 or higher because these levels require therapeutic intervention or infusion interruption. Moreover, IRRs were classified into two groups: nonsignificant IRRs, with no IRRs or IRR grade 1, and significant IRRs, with IRR grades 2−4. Candidate risk factors for IRRs were analyzed for these two groups.

### 2.4. Statistical Analysis

All statistical analyses were performed using the Japanese version of KaleidaGraph^™^ 4 (Hulinks^©^). Comparisons of values of each item were conducted using *t*-, chi-square, or Fisher's exact probability tests. A part of values was expressed as mean (standard deviation (SD)). For all analyses, a *p* value <0.05 was considered statistically significant, and statistical tests were two-sided. Multivariate logistic regression analysis was performed for risk factors that demonstrated a minimum statistical trend (*p* < 0.1) on each comparison of values. The *p* value, odds ratio (OR), and 95% confidence interval (CI) of each risk factor were calculated.

## 3. Results

Overall, 152 patients who received rituximab or a rituximab-containing regimen as the induction therapy during the study period were included in the study. Approximately 25 patients were excluded from the study as they did not fulfil the inclusion criteria, i.e., the second or after time of rituximab infusion therapy (17 patients), no bone marrow aspiration or biopsy test or insufficient results (6 patients), and insufficient laboratory data (2 patients). A total of 127 patients were included in this study, with 43 (34%) patients classified under significant IRRs (IRR grades 2–4). In a cohort of 96 patients without prior glucocorticoid use before rituximab infusion, 38 patients (40%) were classified as significant IRRs. Patient characteristics and clinical data are presented in Table 1. The mean age was 71.7 (11.0) years, and males were found to be slightly predominant (69 patients, 54%). As per the histopathological classification, the most represented type was diffuse large B-cell lymphoma (98 patients, 77%), followed by follicular lymphoma (17 patients, 13%). Using the clinical staging of Ann Arbor classification, 70 (55%) patients were determined to be in the advanced stage of disease (stages III and IV). Thirty-one patients (24%) had used glucocorticoid before undergoing rituximab infusion for the following reasons: to reduce or control tumor volume under equipment of administration of the R-CHOP regimen, to treat another disorder aside from lymphoma, and to begin oral prednisolone intake as a part of the CHOP regimen (under the situation of starting rituximab and CHOP on the same day).

The comparative analysis between nonsignificant IRRs and significant IRRs showed that glucocorticoid use before rituximab infusion was a significant risk-avoiding factor (*p* = 0.016) (Table 1). Advanced stage of disease (stages III and IV) was a risk factor for IRRs in both the all-patient cohort (*p* = 0.018) and the no glucocorticoid premedication cohort (*p* = 0.0058). Bone marrow infiltration of lymphoma cells was found to be a risk factor in the all-patient (*p* = 0.004) and no glucocorticoid premedication (*p* = 0.0007) cohorts. Some trends about splenomegaly were observed as a risk for IRRs in the all-patient cohort (*p* = 0.074). Indolent lymphoma was a trend of risk factor for IRRs in the all-patient cohort (*p* = 0.062). Conversely, elevation of LDH levels to higher than the normal limit, increased sIL-2R levels (>2000 IU/L), and anemia (hemoglobin levels ≤10 g/dL) were not risk factors for grade 2 or higher IRRs. Bulky mass, B symptoms, and ECOG performance status score were also not risk factors in the all-patient and the no glucocorticoid premedication cohorts.

Multivariate analysis in the all-patient cohort identified glucocorticoid administration before rituximab infusion as a strong risk-avoiding factor for IRRs (OR: 0.17; CI: 0.05–0.59; *p* = 0.005). Analysis of the no glucocorticoid premedication cohort identified bone marrow infiltration as a significant risk factor for IRRs (OR: 4.00; CI: 1.02–15.79; *p* = 0.047), which seems to be a more important determining factor than the one based on Ann Arbor staging (Table 2).

## 4. Discussion

A retrospective study was conducted to determine whether bone marrow infiltration is a risk factor under the first induction rituximab therapy in CD20-positive B-NHL. Patients with bone marrow infiltration of B-NHL cells were more likely to be complicated by grade 2 or higher IRRs (15 of 26 patients (58%) in the all-patient cohort and 14 of 19 patients (74%) in the no glucocorticoid premedication cohort). Our result showed that bone marrow infiltration is a risk factor for IRRs in univariate analysis. Additionally, bone marrow infiltration is an independent risk factor for IRRs in the no glucocorticoid premedication cohort in multivariate analysis. Several reports have previously shown that bone marrow infiltration is a risk factor for IRRs in a retrospective study [10, 13, 15], and our result is consistent with these reports. Advanced stage of disease (Ann Arbor staging III or IV) was also found to be a risk factor in univariate analysis. Advanced stage of disease and bone marrow infiltration are speculated to reflect high tumor volume in the body, wherein cytokine release will be more extensive due to the affected lymphoma cells from rituximab. Moreover, bone marrow lymphoma cells may activate surrounding T cells, which are abundant in the bone marrow after the rituximab reaction, resulting in the emergence of more severe IRRs. Patients with bone marrow infiltration of lymphoma cells need to be considered as a high-risk group for IRRs.

In terms of the methods to detect bone marrow lymphoma cells in bone marrow aspiration samples, results of the bone marrow film and FCM analysis were evaluated, and FCM analysis results were more sensitive of the two. A previous study analyzed bone marrow infiltration on the basis of histopathological analysis alone [14, 15] or histopathological as well as FCM analysis [13]. As bone marrow infiltration rate will differ among these methods, both histopathological and FCM analyses will be required for further study.

The association of serum LDH levels (higher than the upper normal limit) and sIL-2R levels (>2000 IU/L) with the risk of IRRs, both of which have no association, has also been investigated. LDH levels are related to the tumor volume of CD20-positive B-NHL, but histopathological type and the speed of tumor growth may also influence serum LDH levels.

Regarding the incidence of IRRs, some variations may emerge because of the glucocorticoid condition or the administrative composition of the rituximab-containing regimen. Kowalski et al. [13] reported the significant risk of CD20-positive B-NHL patients with bone marrow infiltration; 28% of bone marrow-infiltrated patients had IRRs (grades 1–4) under 100 mg hydrocortisone administration. Conversely, our results showed that 74% of bone marrow-infiltrated patients had IRRs (grade 2 or higher) without glucocorticoid premedication. The incidence of IRRs may depend on the status of glucocorticoid premedication.

In this context, glucocorticoid medication on the same day as rituximab infusion might be debatable. Glucocorticoids show immunosuppressive effects and are frequently used to prevent IRRs. Glucocorticoid drug administration just before rituximab infusion or prednisolone intake as a part of the CHOP regimen on the same day of rituximab infusion might reduce the rate and grade of IRRs [12, 17, 18]. Jung et al. suggested that premedication with glucocorticoid should be recommended to patients at high IRR risk at the first rituximab infusion [18]. In our study, rituximab was administered one day before the administration of CHOP or other cytotoxic drugs. For the minority of our patients who received glucocorticoid on the same day of rituximab infusion, the rate of IRRs decreased significantly (Table 1). Previous studies have reported a few rules regarding the date of rituximab infusion in an R-CHOP or R-CHOP-like regimen. Rituximab was administered on the same day of the CHOP regimen in key studies [19–21], and in several studies, rituximab was administered on the day before the CHOP or CHOP-like regimen [22–27]; in some studies, rituximab infusion was acceptable on both of the aforementioned ways [28–30]. Various reasons in practice might influence the manipulation of the rituximab-containing regimen.

Mechanically, complement activation is supposed to be involved in rituximab-related IRRs, and glucocorticoid administration may suppress complement activation. However, complement-mediated cytotoxicity (CMC), which was equipped with rituximab originally, is also suppressed after glucocorticoid administration. In this scenario, it is unclear whether the glucocorticoid premedication affects rituximab's response. Patel et al. reported that grade 2 or higher IRRs are associated with better overall survival in a retrospective analysis of 229 patients with DLBCL who received rituximab [31]. Conversely, Cho et al. showed that IRRs were not associated with OS or progression-free survival in patients with DLBCL [14]. Overall, there is still insufficient evidence of the relationship between IRRs and the response of rituximab [32]. It is likely that uniform glucocorticoid premedication increases the risk of reducing the antitumor effect of rituximab by suppressing CMC; therefore, we adopted the stance that rituximab was administered without glucocorticoid premedication. However, it would be better to perform glucocorticoid premedication to prevent severe IRRs in bone marrow infiltration cases, which is treated as the high-risk group, from a safety perspective.

This study has some limitations. First, this was a retrospective study. Second, the sample size of this study was small, and we did not perform any prespecified power analysis. Third, grading of the IRRs in each case was determined using the description in the EMR; therefore, there may be some variations due as per the attending doctor's judgment. Finally, the rate of IRRs between the histopathological differences in CD20-positive B-NHL was not investigated because of the rather limited sample numbers.

In conclusion, this retrospective study confirmed that bone marrow infiltration of lymphoma cells increases the incidental rate of grade 2 or higher IRRs during rituximab induction therapy without glucocorticoid premedication in CD20-positive B-NHL.

## Figures and Tables

**Table 1:** Patient characteristics and clinical data.

	All patients				Patients without glucocorticoid use before rituximab infusion			
	All	IRR grades 0 and 1	IRR grades 2–4	*p* value	All	IRR grades 0 and 1	IRR grades 2–4	*p* value
Number of Pts	127	84	43		96	58	38	

Age (mean (SD))	71.7 (11.0)	71.3 (11.2)	72.3 (10.4)	^*∗*^0.63	72.6 (10.6)	72.1 (10.5)	73.2 (10.6)	^*∗*^0.66

Gender								
Male	69	46	23	⁺0.89	47	29	18	⁺0.80
Female	58	38	20		49	29	20	

Histopathology								
DLBCL	98	69	29	⁺0.062	70	45	25	⁺0.20
Indolent lymphoma^(a–f)^	29	15	14		26	13	13	
FL^(a)^	17	10	7		16	10	6	
LPL/WM^(b)^	4	2	2		4	2	2	
MCL^(c)^	3	1	2		2	0	2	
SLL^(d)^	2	0	2		2	0	2	
SMZL^(e)^	2	1	1		1	0	1	
MALT^(f)^	1	1	0		1	1	0	

Ann Arbor staging								
I/II	57	44	13	⁺0.018	47	35	12	⁺0.0058
III/IV	70	40	30		49	23	26	

ECOG PS								
2 or more	108	71	37	⁺0.82	10	5	5	^‡^0.51
0, 1	19	13	6		86	53	33	

sIL-2R (U/mL)								
>2000	33	24	9	⁺0.74	18	12	6	⁺0.94
≤2000	79	55	24		67	44	23	

LDH (U/L)								
>ULN	58	37	21	⁺0.61	36	18	18	⁺0.11
≤ULN	69	47	22		60	40	20	

Hemoglobin (g/dL)								
>10	102	67	35	⁺0.83	79	50	29	⁺0.21
≤10	25	17	8		17	8	9	

Bone marrow infiltration								
Present	26	11	15	⁺0.004	19	5	14	⁺0.0007
Absent	101	73	28		77	53	24	

Splenomegaly								
Present	17	8	9	⁺0.074	9	3	6	^‡^0.149
Absent	110	76	34		87	55	32	

B symptoms								
Present	17	12	5	⁺0.68	4	1	3	^‡^0.30
Absent	110	72	38		92	57	35	

Bulky mass								
Present	9	6	3	^‡^1.0	9	6	3	^‡^1.0
Absent	118	78	40		87	52	35	

Circulating lymphoma cells (/*μ*l)								
>25000	1	0	1	^‡^0.34	1	0	1	^‡^0.40
≤25000	126	84	42		95	58	37	

Number of previous chemotherapy regimens								
0	127	84	43	^‡^1.0	96	58	38	^‡^1.0
≥1	0	0	0		0	0	0	

Glucocorticoid use before rituximab infusion								
Present	31	26	5	⁺0.016	—	—	—	
Absent	96	58	38		—	—	—	

Statistical analysis, ^*∗*^*t*-test, ^⁺^chi-square test, and ^‡^Fisher's exact test. IRRs: infusion-related reactions; DLBCL: diffuse large B-cell lymphoma; FL: follicular lymphoma; MCL: mantle cell lymphoma; MALT: mucosa-associated lymphoid tissue lymphoma; LPL: lymphoplasmacytic lymphoma; WM: Waldenström macroglobulinemia; SLL: small lymphocytic lymphoma; SMZL: splenic marginal zone lymphoma; sIL-2R: serum interleukin-2 receptor; LDH: lactate dehydrogenase; ULN: upper limit of the normal range; ECOG PS: Eastern Cooperative Oncology Group performance status score; SD: standard deviation; Pts: patients. Indolent lymphoma was composed of FL, LPL/WM, MCL, SLL, SMZL, and MALT.

**Table 2:** Multivariate logistic regression analysis.

Group	Factor	Odds ratio	95% confidence interval	*p* value
All patients	Ann Arbor staging (III and IV)	2.09	0.84–5.24	0.118
	Bone marrow infiltration	2.03	0.56–7.43	0.284
	Splenomegaly	1.81	0.44–7.50	0.415
	Indolent lymphoma	0.87	0.29–2.61	0.800
	Glucocorticoid use before rituximab infusion	0.17	0.05–0.59	0.005

Patients without glucocorticoid use before rituximab infusion	Ann Arbor staging (III and IV)	1.94	0.73–5.18	0.185
	Bone marrow infiltration	4.00	1.02–15.79	0.047

## Data Availability

The dataset used in this study is available in our institutionalized database.
